# Prior Knowledge and Integrated Metacognitive Support in Hypermedia Learning: Effects on Self-Regulated Learning Processes and Outcomes

**DOI:** 10.3390/bs16071200

**Published:** 2026-07-16

**Authors:** Chenyin Wang, Yanqing Chen, Guoxia Wang

**Affiliations:** School of Psychology, Northeast Normal University, Changchun 130024, China; wangcy331@nenu.edu.cn (C.W.); 18088689314@163.com (Y.C.)

**Keywords:** hypermedia learning, self-regulated learning, metacognitive support, prior knowledge

## Abstract

Hypermedia learning environments place high demands on learners’ self-regulation, yet many learners struggle to plan, monitor, and evaluate their understanding. This study examined whether integrated metacognitive support improves self-regulated learning (SRL) processes and learning outcomes, and whether these effects differ by learners’ prior knowledge. A 2 × 2 between-subjects design was used with 103 undergraduates learning about the human circulatory system in hypermedia learning environments. Participants were randomly assigned to an integrated metacognitive support condition or a control condition, and prior knowledge was measured before learning. Results showed that integrated metacognitive support significantly increased planning, monitoring, rehearsal, elaboration, and organization, and improved retention, comprehension, overall post-test scores, and proportional learning gains. Prior knowledge did not moderate support effects on SRL strategy frequencies, but process analyses revealed distinct SRL patterns across prior knowledge groups. In particular, the support helped learners with low prior knowledge shift toward a more adaptive regulatory pattern, while mainly strengthening the existing regulatory pattern among learners with high prior knowledge. Learners with low prior knowledge also benefited more from integrated support in comprehension and overall post-test scores. These findings indicate that integrated metacognitive support can strengthen SRL in hypermedia environments, particularly for learners with limited prior knowledge. Importantly, the results suggest that these effects extend beyond strategy use to learning processes during hypermedia learning. Accordingly, metacognitive scaffolds in hypermedia learning systems should be designed with learners’ prior knowledge in mind.

## 1. Introduction

Hypermedia learning environments provide learners with flexible access to information through text, images, videos, and non-linear navigation (Moos & Marroquin, 2010). This flexibility can support active learning, but it also places high demands on learners’ self-regulation. Learners need to clarify task demands, set goals, choose appropriate strategies, monitor the effectiveness of these strategies, and evaluate their emerging understanding (Azevedo, 2005). However, many learners do not spontaneously engage in effective self-regulated learning (SRL) in hypermedia environments (Azevedo, 2009; Bannert et al., 2015; Zimmerman, 2008). They may read passively, fail to monitor their understanding, and/or continue using ineffective strategies when difficulties arise. For this reason, researchers have designed different forms of instructional support to help learners regulate their learning more effectively.

Metacognitive support is one of the most widely used approaches for supporting SRL in hypermedia learning (Azevedo, 2005; Bannert & Reimann, 2012; Bannert et al., 2015). Such support often prompts learners to plan, monitor, evaluate, and reflect during learning. Prior studies have shown that metacognitive supports can improve learners’ SRL activities and their learning performance (L. Guo, 2022; Zheng, 2016). Alongside the intervention research, a growing body of process-oriented SRL research has examined the process patterns of regulatory activities using methods such as process mining, sequential analysis, and more recently network analysis (Bannert et al., 2014; Fan et al., 2023; Malmberg et al., 2017; Sonnenberg & Bannert, 2015; Yang et al., 2018). By examining how learning activities unfold over time, these process-oriented studies suggest that the effect of metacognitive support is not only reflected in whether learners use planning, monitoring, or evaluation more frequently, but also in how these activities are organized and connected during learning.

However, the effectiveness of metacognitive support may depend on how it is designed. Metacognitive support may be more effective when it addresses both metacognitive skills and metacognitive knowledge (Bannert & Reimann, 2012; Donker et al., 2014; Zepeda et al., 2019). Learners may be encouraged to engage in regulatory activities, but they also need to understand why these activities are useful and how they should be applied during learning. This suggests that metacognitive support may be particularly useful when it helps learners both understand SRL activities and carry them out during hypermedia learning.

However, learners may not respond to metacognitive support in the same way. Prior knowledge is an important learner characteristic in hypermedia learning. Learners with higher prior knowledge (HPK) may be better able to define task demands, judge information relevance, and engage in planning and monitoring activities during hypermedia learning, whereas learners with lower prior knowledge (LPK) may struggle with these judgments and rely on a narrower set of strategies (Moos & Azevedo, 2008). From an aptitude-treatment interaction perspective, the effectiveness of instructional support may depend on learners’ prior knowledge (Koran & Koran, 1984). Instructional support may make a greater difference when learners have less prior knowledge of the learning content; when learners have more prior knowledge, differences between instructional conditions may become less pronounced. This suggests that prior knowledge may influence the effects of metacognitive support.

Previous studies have examined the role of prior knowledge in hypermedia SRL from two related perspectives. First, studies examining SRL among learners with high and low prior knowledge have found differences in cognitive and metacognitive strategy use, navigation behavior, and learning performance (Moos & Azevedo, 2009; Taub et al., 2014; Taub & Azevedo, 2019). Second, other studies have examined whether prior knowledge influences the effects of instructional support (Bannert & Reimann, 2012; Trevors et al., 2014). For example, Trevors et al. (2014) found that metacognitive intervention reduced ineffective note-taking strategies among LPK learners but had no significant effect on those with HPK. These studies have advanced our understanding of how prior knowledge relates to instructional support and to SRL processes. However, few studies have used a factorial design that includes both support and control conditions for high and low prior knowledge learners. In addition, whether prior knowledge influences the effects of metacognitive support on SRL process patterns has rarely been examined directly.

Addressing this gap matters because the same regulatory activity may serve different functions depending on its position in the learning process (Fan et al., 2023). The present study addressed this gap by examining the effects of integrated metacognitive support and prior knowledge on learners’ SRL processes and learning outcomes during hypermedia learning. The present study uses the term integrated metacognitive support to refer to an approach that combines pre-learning metacognitive instruction and metacognitive prompts and feedback embedded in the learning task. The study used a 2 × 2 between-subjects design, with support and control conditions for both LPK and HPK learners. Think-aloud protocols and screen recordings were collected, and the data were analyzed using factorial ANOVAs and ordered network analysis (ONA; Tan et al., 2022). The design allowed us to examine whether integrated metacognitive support had different effects on LPK and HPK learners across strategy frequency, process pattern, and learning outcomes. By combining frequency-based and process-based analyses, this study examined not only whether the support increased SRL strategy use but also whether it influenced how learners regulated their learning during hypermedia learning. The findings may inform the design of hypermedia learning environments that provide more targeted guidance based on learners’ prior knowledge. Accordingly, the study addresses the following research questions:

**RQ1.**  *How does integrated metacognitive support influence learners’ SRL strategy frequencies, and how do these effects differ between learners with low and high prior knowledge?*

**RQ2.**  *How does integrated metacognitive support influence the sequential structure of learners’ SRL processes, and how do these patterns differ between learners with low and high prior knowledge?*

**RQ3.**  *How does integrated metacognitive support influence learners’ learning outcomes, and how do these effects differ between learners with low and high prior knowledge?*

### 1.1. Metacognitive Support for Self-Regulated Learning

Metacognition is commonly understood as involving both learners’ knowledge about cognition and their regulation of cognition (Flavell, 1979; Schraw, 2001; Veenman et al., 2006). Following Veenman et al. (2006), the present study distinguishes between metacognitive knowledge and metacognitive skills. Metacognitive knowledge refers to learners’ knowledge about themselves, task demands, and strategy use, whereas metacognitive skills refer to the processes through which learners plan, monitor, and evaluate their learning (Veenman et al., 2006). Although these two components can be distinguished conceptually, both are important for effective SRL. Winne and Hadwin (1998) conceptualized SRL as a recursive and weakly sequenced process involving task definition, goal setting and planning, enactment, and adaptation. Across these stages, learners may draw on metacognitive knowledge to interpret task demands, set goals, and guide strategic choices, while metacognitive skills enable them to monitor, control, and adapt learning as it unfolds.

A substantial body of research suggests that metacognitive support can improve learners’ SRL and learning outcomes in hypermedia environments. In a meta-analysis, L. Guo (2022) found that metacognitive prompts had moderate positive effects on both learners’ SRL activities and learning outcomes. However, prompting learners to regulate may not be sufficient on its own. As Pintrich (2002) argued, learners who possess greater knowledge about strategies for learning, thinking, and problem solving are more likely to use them effectively. Without such knowledge, learners may be prompted to monitor or evaluate their learning without understanding what to focus on, which strategies are appropriate, or when particular strategies should be used. Moreover, metacognitive knowledge and metacognitive regulation are closely related and may support one another (Schraw, 2001). This interdependence may be especially salient in hypermedia environments, where insufficient metacognitive knowledge may constrain learners’ ability to select and apply metacognitive skills effectively.

Empirical evidence is consistent with this view. In hypermedia learning, Bannert and Reimann (2012) found that metacognitive prompting combined with training produced stronger effects on learners’ transfer performance than prompting alone. Related evidence from broader educational settings also highlights the value of addressing both metacognitive knowledge and metacognitive skills in instructional support (Donker et al., 2014; Zepeda et al., 2019). These findings suggest that metacognitive support in hypermedia environments may be more effective when it addresses both components.

Building on this perspective, the present study adopts an integrated metacognitive support approach that combines pre-learning instruction on metacognitive knowledge and skills with embedded metacognitive prompts and feedback during the hypermedia learning task. The specific design of the support is described in Section 2.4.

### 1.2. Prior Knowledge and Self-Regulated Learning

Prior knowledge is an important cognitive condition in hypermedia learning because it influences how learners understand the task and regulate their learning. This view is consistent with Winne and Hadwin’s (2008) account of SRL, in which learners construct a personalized understanding of the task based on external task conditions and internal conditions, including knowledge retrieved from long-term memory. Within this framework, prior knowledge may influence how learners interpret task demands, identify relevant information, select possible operations, and judge what counts as satisfactory progress. Its role may be particularly salient in hypermedia environments, where learners must navigate non-linearly, coordinate multiple information sources, and decide what information is worth attending to (Moos & Azevedo, 2008).

Empirical research has shown that learners who differ in prior knowledge regulate their learning differently in hypermedia environments. Using think-aloud data, Moos and Azevedo (2008) found that prior domain knowledge was positively related to planning and monitoring activities but negatively related to the use of cognitive strategies. In a related study, Moos and Azevedo (2009) further found that prior knowledge was significantly related to learners’ monitoring of understanding and learning outcomes. These findings suggest that prior knowledge affects not only what learners know before a task, but also how they engage in regulatory activities and how they perform after hypermedia learning.

Further, prior knowledge may also influence how learners benefit from instructional support. According to the expertise reversal effect, support that is helpful for LPK learners may become redundant or even interfere with learning for HPK learners, who can rely more on existing knowledge structures (Kalyuga, 2007). Some empirical findings are consistent with these expectations. Trevors et al. (2014) reported an interaction between prior knowledge and scaffolding: LPK learners took more notes than HPK learners only when scaffolding was absent, whereas the two groups showed comparable note-taking when scaffolding was provided. Similarly, Taub and Azevedo (2019) found that HPK learners showed smaller proportional learning gains compared to LPK learners after learning with a multi-agent hypermedia system. Yang et al. (2018) reported that, after extended use of an SRL support system, the performance gap between LPK and HPK learners was no longer significant in a delayed test. These findings indicate that prior knowledge may influence the effectiveness of metacognitive support.

Although these findings are informative, the existing evidence remains limited in two respects. First, many studies have relied mainly on learning outcomes or comparisons within a single support condition, without including a no-support control group (e.g., Yang et al., 2018). Other studies have examined differences in prior knowledge, rather than directly testing whether prior knowledge moderates the effects of support on SRL process patterns (e.g., Taub & Azevedo, 2019). Second, studies that have compared support and control conditions across prior knowledge groups have usually focused on selected strategies (e.g., Trevors et al., 2014). Less is known about how metacognitive support affects the broader range of SRL strategies and the temporal relations among these strategies. To address these limitations, a factorial design is needed to compare the effects of metacognitive support across prior knowledge groups. Process-oriented analyses are also needed to capture how SRL strategies unfold over time. The next section reviews analytic approaches for examining SRL process data.

### 1.3. Process Analysis of Self-Regulated Learning

Advances in learning analytics have made it possible to examine SRL as a temporal and connected process rather than as a set of isolated activities. Compared with traditional frequency-based analysis, process analysis provides richer information about how learning activities unfold over time and how different actions are linked during learning. Accordingly, approaches such as process mining, sequential mining, and ONA have been used to identify temporal patterns of learning behaviors (Q. Guo et al., 2026; He et al., 2024; T. Li et al., 2023; Taub & Azevedo, 2019).

This perspective is important for SRL. Theoretically, SRL is not defined simply by whether learners engage in planning, monitoring, or strategy use, but how these activities are connected and adapted during learning. Empirical studies have shown that successful learners showed more regulation event types, such as frequent monitoring and evaluation activities, whereas less successful learners typically relied on a surface approach to learning, with preparatory and evaluation activities partly missing (Bannert et al., 2014). Similarly, Lim et al. (2021) found that successful learners showed links among cognitive and metacognitive activities, as well as between superficial and deep cognitive activities, whereas less successful learners had difficulty combining them, with monitoring disconnected from deeper cognitive activities and evaluation disconnected from analysis.

Research also shows that learners’ SRL processes are influenced by metacognitive supports. Sonnenberg and Bannert (2015) found that orientation, planning, and goal specification were only weakly connected to the overall learning process in the control group but were well embedded in the learning processes of students who received metacognitive support. These students also showed more loops between metacognitive and cognitive events. More recently, Engelmann and Bannert (2021) found that although the overall learning processes of students with and without metacognitive prompts were generally similar, evaluation was better integrated into the learning process among students who received metacognitive prompts. These findings indicate that metacognitive support may influence how regulatory activities unfold during learning.

Prior knowledge also appears to affect how learners engage in SRL during hypermedia learning. Using differential sequence mining analysis, for instance, researchers discovered that HPK learners tended to use metacognitive strategies before cognitive strategies, whereas LPK learners tended to use cognitive strategies before metacognitive strategies (Taub et al., 2014). Likewise, Taub and Azevedo (2019) reported that HPK learners were more likely to engage in sequences containing both cognitive and metacognitive SRL processes, whereas LPK learners were less likely to display such patterns. However, prior research has not sufficiently examined the interaction between prior knowledge and metacognitive support from a process-oriented perspective.

To examine this issue, process analyses are needed because they can show how SRL activities unfold during learning. However, different analytic approaches capture different aspects of learning processes. More recently, ordered network analysis (ONA) has been used to examine learning processes by modeling directed connections among learning activities and capturing their temporal and structural relations (Tan et al., 2022). In addition, Fan et al. (2023) showed that, compared with process mining, ONA can reveal not only the frequency, continuity, and sequentiality of actions, but also their relative roles within a broader pattern. Recent studies have also used ONA to compare learners’ regulatory processes across different support conditions and have shown that different forms of support can be associated with different regulatory pathways (Q. Guo et al., 2026).

In the present study, ONA is used to examine whether integrated metacognitive support changes not only how often learners engage in SRL activities, but also how these activities unfold during learning, and whether these patterns differ between HPK and LPK learners.

## 2. Materials and Methods

### 2.1. Participants and Experimental Design

Participants were 103 undergraduate students from non-biology disciplines, including Mathematics, History, Education, Chinese Language and Literature, Physics, Chemistry, Philosophy, Psychology, Geography, Physical Education, and Marxist Theory. Of these participants, 80.6% were female. The study used a 2 × 2 between-subjects design with instructional condition and prior knowledge as the two factors. Instructional condition was experimentally manipulated, with participants randomly assigned to either the integrated metacognitive support condition (*n* = 51, 80.4% female) or the control condition (*n* = 52, 80.8% female). Prior knowledge was measured before learning and then dichotomized using a median split for group-based analyses. Participants with scores at or below the median were assigned to the LPK group (*n* = 58, 77.6% female), whereas those with scores above the median were assigned to the HPK group (*n* = 45, 84.4% female). Because prior knowledge was measured rather than manipulated, cell sizes were unequal: support–HPK (*n* = 25), support–LPK (*n* = 26), control–HPK (*n* = 20), and control–LPK (*n* = 32). Think-aloud data were unavailable for four participants because of recording failure. All four participants were in the LPK group; therefore, analyses based on think-aloud data included 99 participants.

### 2.2. Learning Environment

**Figure 1:**
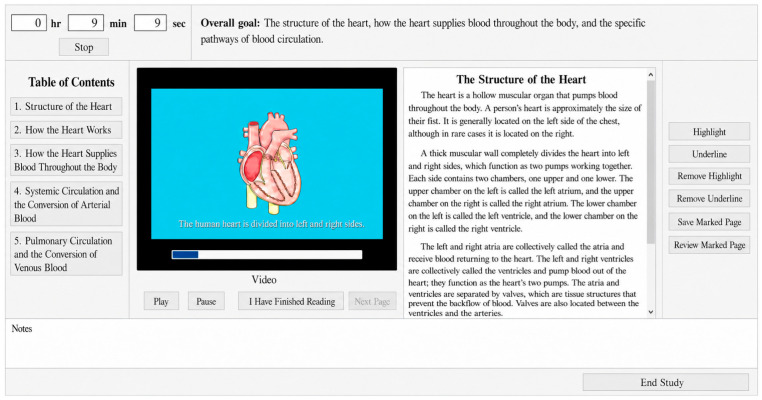
Screenshot of the hypermedia learning environment for the control condition.

**Figure 2:**
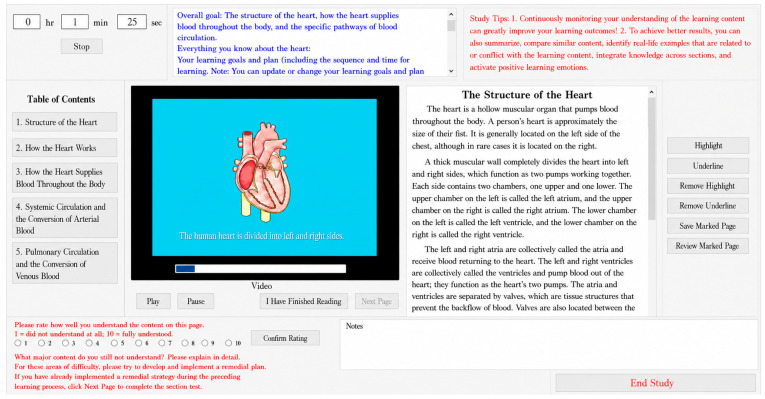
Screenshot of the hypermedia learning environment for the experimental condition.

Two hypermedia learning environments were developed for this study: one for the control condition and one for the integrated metacognitive support condition (see Figure 1 and Figure 2, respectively). Both environments provided the same learning materials on the cardiovascular system, comprising 1471 words of text, two images, three videos, and five tests organized into five sections.

The two environments shared the same basic interface and functions. Each included (1) a timer for monitoring time on task, (2) a learning goals box, (3) a table of contents for navigation, (4) a media area displaying videos or images, (5) a text area presenting content related to the media, (6) a toolbar with annotation tools such as highlighting and underlining, and (7) a notes box below the content area for recording thoughts during learning. These note-taking, highlighting, and underlining tools were provided as general interface functions commonly used in hypermedia learning environments. Similar interface features, such as note-taking and highlighting, have also been included in prior hypermedia learning studies to support learners’ interaction with learning materials (e.g., Fan et al., 2022). These tools allowed learners to mark or record information during reading and were available in both conditions; therefore, they were not part of the metacognitive support manipulation. Learners were required to complete the test for each section before advancing to the next. They could progress through the materials sequentially or move across sections using the table of contents.

Compared with the control environment, the support environment included additional features designed to promote SRL. First, the learning goals box prompted learners to activate prior knowledge, set subgoals, and make plans. Second, a “Study Tips” box was displayed in the upper-right corner of the screen to encourage metacognitive strategy use, such as comprehension monitoring and connecting new information to prior knowledge. Third, after each content page, learners were prompted to complete self-assessment and reflection questions shown at the bottom of the screen. These prompts asked learners to evaluate their understanding, identify difficulties, and consider possible remedial strategies. Finally, after each section test, learners received feedback on the correct answers along with metacognitive knowledge support, which was intended to guide learners’ monitoring and regulation of their subsequent learning.

### 2.3. Material and Instruments

#### 2.3.1. Measuring Learner Characteristics

Academic self-concept was measured using 5 items adopted from PISA 2012 (OECD, 2014). The scale was adapted by Chinese researchers and has shown good reliability and validity (Kong, 2015). Learners were asked to answer on a 5-point Likert scale (1 = not at all true of me, 5 = very true of me). A sample item was: “I’m good at learning.” Reliability for the scale was good (α = 0.75).

Self-regulated learning strategies were measured using the cognitive strategy use and self-regulation subscales of the Motivated Strategies for Learning Questionnaire (MSLQ; Pintrich & De Groot, 1990). The two subscales included 22 items in total. Learners were asked to answer on a 7-point Likert scale (1 = not at all true of me, 7 = very true of me). A sample item from the self-regulation subscale was: “I work hard to get a good grade even when I don’t like a class.” Reliability for the scale was good (α = 0.81).

Learners’ metacognition was assessed using a 14-item instrument developed by combining items from the Metacognitive Awareness Inventory (MAI; Schraw & Dennison, 1994), the Awareness of Independent Learning Inventory (AILI; Meijer et al., 2013), and the Offline Metacognitive Moderation Questionnaire (J. Li et al., 2012). This combined instrument has been adopted by researchers in China and has shown good reliability and validity (Kong, 2015). The items assessed metacognitive knowledge, metacognitive skills, and metacognitive experience. Learners were asked to answer on a 5-point Likert scale (1 = not at all true of me, 5 = very true of me). A sample item was: “I can skillfully apply some useful learning methods.” Reliability for the scale was good (α = 0.76).

#### 2.3.2. Measuring SRL Process

Think-aloud protocols and video data were recorded during the learning session using a two-step procedure. First, the data were divided into meaningful segments, then each segment was given a unique code. Two trained researcher assistants independently conducted blind coding of the verbal protocols from ten participants to establish inter-rater reliability, which showed high agreement (Cohen’s kappa = 0.91). Any discrepancies were subsequently discussed and resolved through consensus.

Think-aloud data were coded using a hypermedia SRL coding scheme adapted from Azevedo and Cromley (2004) and Glogger et al. (2012). As shown in Table 1, the scheme included seven categories: Planning, Monitoring, Reading, Rehearsal, Elaboration, Organization, and Test. The first six categories captured learners’ SRL activities during hypermedia learning. Test was included as an additional category because learners were required to complete an embedded assessment at the end of each chapter before proceeding to the next chapter.

#### 2.3.3. Measuring Prior Knowledge and Performance

Learners’ prior knowledge was assessed using a pre-test composed of a retention test and a comprehension test. The same set of items was also administered as the post-test. The retention test consisted of 10 multiple-choice items, each with one correct answer. The comprehension test consisted of multiple-choice questions, some with one correct answer and some with multiple correct answers, as well as two open questions. All items assessed knowledge of the domain to be learned (e.g., “the circulation of the body begins”; open question: “when a person injects a drug during an illness, in which chamber of the heart is the drug first found if the person is traced”). The maximum possible score was 23 (retention = 10, comprehension = 13). The reliability of the pre-test was acceptable (McDonald’s ω = 0.71).

Four variables were created to assess learners’ performance: retention scores, comprehension scores, overall post-test scores, and proportional learning gains (The formula is: (PostRatio − PreRatio)/(1 − PreRatio) (Taub & Azevedo, 2019)). These indicators were used to provide a more comprehensive evaluation of learning performance. The reliability of the post-test was good (McDonald’s ω = 0.88).

### 2.4. Integrated Metacognitive Support

Participants in the integrated metacognitive support condition received support both before and during the hypermedia learning session. Before learning, they were provided with structured instructional materials introducing SRL, metacognitive knowledge, and metacognitive skills. These materials explained that effective learners tend to set learning goals, select learning strategies, seek relevant resources, monitor learning progress, adjust plans and actions, and evaluate goal attainment after learning. They also introduced metacognitive knowledge as knowledge about the self, the task, and strategy use, and explained metacognitive skills in terms of planning, monitoring, evaluation, and reflection. Each concept was illustrated with everyday examples. For instance, to explain task knowledge, the materials described a student who reviews an exam paper, identifies familiar and unfamiliar topics, and plans which questions to attempt first. To explain monitoring, the materials described a student who notices that too much time has been spent on one question and adjusts the plan to prioritize questions worth more points. The materials also introduced common cognitive strategies, including marking key information, note-taking, summarizing, comparing similar content, and connecting new information to prior experience.

During the learning session, this pre-learning support was supplemented by metacognitive prompts embedded in the learning environment. Before beginning the learning task, the learning goals box prompted learners to activate prior knowledge, set subgoals, and make a study plan. During learning, a “Study Tips” box reminded learners to monitor their comprehension continuously and to use strategies such as summarizing, comparing related content, and connecting new information to prior knowledge. After each content page, learners completed self-assessment and reflection prompts. Specifically, they were asked to rate their understanding of the page on a scale from 1 (did not understand at all) to 10 (fully understood), describe any content they still found unclear, and make a remedial plan to address those difficulties before proceeding to the section test. After each section test, they received feedback on the correct answers together with metacognitive knowledge support. Taken together, the support was designed to facilitate planning before learning, monitoring and reflection during learning, and evaluation and adjustment after task performance. In contrast, participants in the control condition received reading materials unrelated to metacognition before learning and completed the learning session without these metacognitive prompts, feedback, or strategy-related guidance.

### 2.5. Procedures

**Figure 3:**
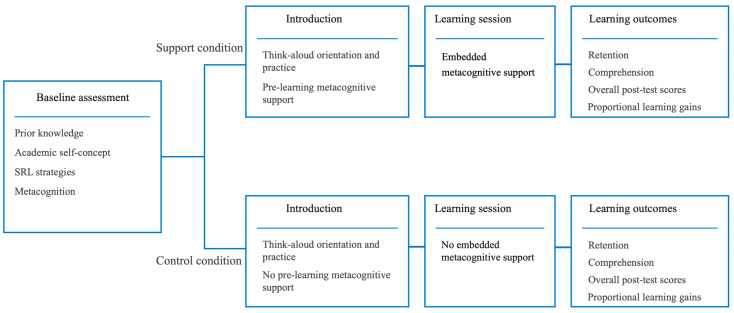
Procedure of the study.

Figure 3 illustrates the study procedure. First, participants completed an online baseline assessment measuring their prior knowledge and several learner characteristics, including academic self-concept, SRL strategies, and metacognition. This phase took approximately 20 min.

Next, participants were introduced to the learning environment and to the think-aloud procedure through a video that featured the same learning environment as the formal experiment. After that, participants engaged in a 5-min think-aloud practice session using instructional materials on the formation of lightning. This practice aimed to ensure participants understood the think-aloud procedure and could apply it effectively during the learning task.

After the practice phase, learners in the metacognitive support condition received the instructional materials described in Section 2.4, while those in the non-metacognitive support condition read materials unrelated to metacognition. They then engaged in the hypermedia learning task on the topic of heart and blood circulation, with a total learning time of approximately 40 min. The learning objectives were displayed on-screen throughout the task. Learners in the metacognitive support condition were prompted to activate prior knowledge, set subgoals, and make a learning plan before learning, and they received self-assessment, reflection, and feedback support during the learning session. Participants in the control condition completed the same learning task without these supports.

Immediately after the learning session, all participants completed a post-test assessing their understanding of the learning content. Throughout the hypermedia learning, participants were free to navigate the environment and were required to think aloud continuously. Their verbalizations and on-screen activities were recorded for subsequent analysis. Participants were informed that they would complete a learning test after the hypermedia learning task. However, they were not told the specific test format or test items, so as to avoid test-specific preparation during learning.

### 2.6. Data Analysis

Different analytic approaches were used to address the research questions. To address RQ1 and RQ3, a series of two-way factorial ANOVAs were conducted to examine the main and interaction effects of integrated metacognitive support and prior knowledge on SRL strategies and learning outcomes. To address RQ2, we used ONA to examine the temporal and structural characteristics of learners’ SRL processes.

Several learner characteristics were considered as potential covariates. Correlation analyses indicated that these variables had limited prognostic value for the posttest outcomes. Because the value of covariate adjustment depends primarily on the prognostic strength of the covariates (Stallasch et al., 2024), their inclusion was unlikely to improve estimation precision. In addition, although some baseline differences were observed, baseline significance tests are not recommended for selecting covariates in randomized trials (De Boer et al., 2015). Therefore, these learner characteristics were not included as covariates in the analysis, in order to maintain model parsimony.

For the factorial ANOVAs, the assumptions of linearity and independence of errors were satisfied, and multicollinearity was not a concern (all VIFs < 3). However, several models showed non-normal residuals, and some violated the homoscedasticity assumption. Recent simulation evidence indicates that heteroskedasticity-consistent inference methods, particularly HC3 and HC4, often provide more trustworthy results than conventional procedures when residuals are non-normal or heteroskedastic, and that HC4 may be especially advantageous in finite samples with influential observations (Rajh-Weber et al., 2025). Therefore, although the 2 × 2 factorial ANOVAs were estimated in the ordinary least squares framework, hypothesis testing was based on HC4-robust Type III ANOVA. Effect sizes are reported as partial eta squared.

For the process data, ONA was used to model directed connections among SRL behaviors and to examine patterns of SRL processes across conditions. ONA is appropriate when the order of events is meaningful because it models directed relations among coded behaviors within a local temporal context and supports both visual and statistical comparisons of networks (Tan et al., 2022). Based on the coded SRL behaviors, we constructed directed weighted networks and compared mean networks across prior knowledge and support conditions.

## 3. Results

### 3.1. Effects of Integrated Metacognitive Support and Prior Knowledge on Self-Regulated Learning Strategies

To address the first research question, we analyzed the coded think-aloud protocols to examine the effects of integrated metacognitive support and prior knowledge on learners’ SRL strategies. Means (standard deviations) for each SRL strategy in all groups are presented in Table 2.

Table 3 shows the HC4-robust Type III ANOVA results for the SRL strategies. Overall, the support effect was statistically significant for all five SRL strategies. Compared with learners in the control condition, learners in the support condition engaged more frequently in planning, monitoring, rehearsal, elaboration, and organization. The strongest effect was observed for monitoring. In contrast, the main effect of prior knowledge was not significant for any SRL strategies, although elaboration showed a marginal trend. Likewise, the interaction between support and prior knowledge was not significant for any SRL strategies. These findings suggest that the integrated metacognitive support had a broad positive effect on learners’ use of SRL strategies, whereas prior knowledge contributed little to explaining differences in these coded strategies.

### 3.2. Effects of Integrated Metacognitive Support and Prior Knowledge on Self-Regulated Learning Process Patterns

**Figure 4:**
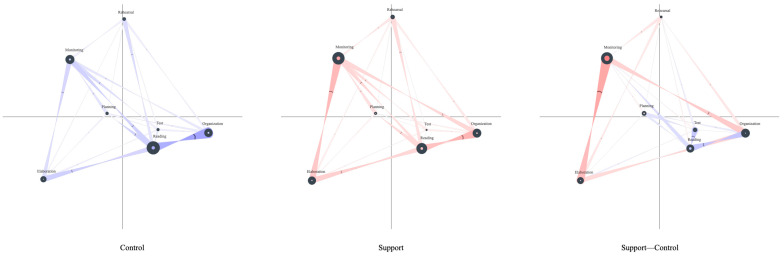
ONA networks for LPK learners in the control and support conditions and their subtraction network (left to right). Blue represents the control condition and red represents the support condition; in the subtraction network, each color indicates stronger connections in the corresponding condition.

**Figure 5:**
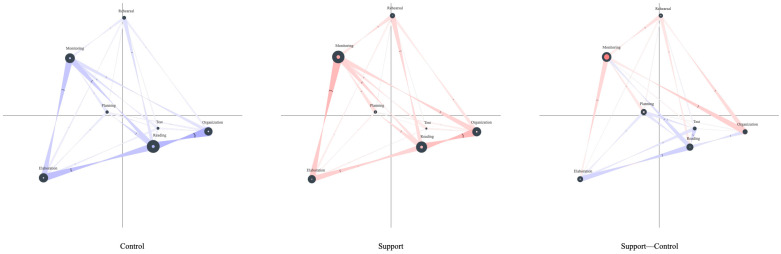
ONA networks for HPK learners in the control and support conditions and their subtraction network (left to right). Blue represents the control condition and red represents the support condition; in the subtraction network, each color indicates stronger connections in the corresponding condition.

To address the second research question, we used ONA to examine learners’ SRL process patterns across conditions and visualized the networks separately within each prior knowledge group. As depicted in Figure 4 and Figure 5, node size reflects the relative frequency of a behavior as a response, the inner circle indicates self-transitions, and edge thickness reflects the relative strength of directed connections. In the subtraction networks, blue edges represent relatively stronger connections in the control group, whereas red edges represent relatively stronger connections in the intervention group.

For LPK learners, the intervention and control conditions differed significantly on both dimensions of the ONA embedding space: Dimension 1, Welch’s *t*(52.00) = 2.48, *p* = 0.017, and Dimension 2, Welch’s *t*(51.79) = −2.64, *p* = 0.011. Specifically, the control group had a higher mean score on Dimension 1 (*M* = 0.132, *SD* = 0.327) than the intervention group (*M* = −0.073, *SD* = 0.279), whereas the intervention group had a higher mean score on Dimension 2 (*M* = 0.069, *SD* = 0.162) than the control group (*M* = −0.061, *SD* = 0.201). These results indicate that LPK learners in the two conditions showed distinct SRL process patterns.

As shown in Figure 4, learners in the control group showed a clear reading-centered pattern. Reading was the most prominent node and also showed relatively strong self-transition. In terms of edge structure, transitions commonly moved from planning to reading, and from reading to organization, rehearsal, and elaboration. This pattern suggests that learners often began by reading new material and then engaged in activities such as repeating, taking notes, summarizing, or linking the material to prior knowledge. The network also showed transitions from elaboration, test, rehearsal, and organization to monitoring, as well as transitions from monitoring back to reading. This suggests that learners monitored and evaluated their understanding during learning, but these activities were often followed by continued reading rather than by further strategic adjustment.

In contrast, in the intervention group, monitoring became the most prominent node and showed a stronger self-transition, indicating more frequent and sustained monitoring of progress, strategy use, information adequacy, and task difficulty. The intervention group showed transitions from monitoring to organization and rehearsal, suggesting that after monitoring their progress and evaluating their current understanding, learners were more likely to revisit previously learned content and reorganize the material. The subtraction network further showed relatively stronger self-transitions in monitoring, together with stronger links among monitoring, rehearsal, organization, and elaboration in the intervention condition. By contrast, the control group showed relatively stronger self-transitions in reading, as well as stronger transitions from organization to reading, reading to test, and from monitoring back to reading. Thus, although learners in the control group also engaged in monitoring and organization, these activities more often led back to continued reading than to subsequent cognitive strategies for regulating learning. These patterns suggest that, among LPK learners, the intervention was associated with a shift away from a reading-centered pattern toward stronger links between monitoring and subsequent cognitive activities.

For HPK learners, the intervention and control conditions did not differ significantly on Dimension 1 of the ONA embedding space, Welch’s *t*(34.33) = 0.75, *p* = 0.460. However, they differed significantly on Dimension 2, Welch’s *t*(40.33) = −4.02, *p* < 0.001. The intervention group had a higher mean score on Dimension 2 (*M* = 0.094, *SD* = 0.170) than the control group (*M* = −0.114, *SD* = 0.175). These results suggest that HPK learners also showed differences in SRL process patterns, but these differences were mainly reflected on Dimension 2 rather than across both dimensions.

As shown in Figure 5, learners in the control group already displayed a relatively rich SRL network. Reading, monitoring, organization, and elaboration were all prominent nodes, with reading remaining the largest node and showing a relatively strong self-v. The network also showed several links among cognitive strategies, including transitions from organization to elaboration and rehearsal, and from rehearsal to elaboration. In addition, a transition from monitoring to rehearsal was also visible. This pattern suggests that HPK learners in the control condition showed a clear regulatory sequence, in which monitoring and evaluation were followed by subsequent cognitive strategy use.

The intervention group showed a broadly similar overall pattern to the control group. The main difference was that, in the intervention group, monitoring became the most prominent node and showed a stronger self-transition. In addition, a transition from monitoring to organization was also visible. The subtraction network also indicated relatively stronger links in the intervention condition among rehearsal, organization, and elaboration, as well as stronger links from monitoring to subsequent cognitive strategy use. By contrast, the control group showed relatively stronger paths that returned to reading, test, or planning. These results suggest that, among HPK learners, the intervention did not change the overall SRL process pattern but made monitoring more prominent and more frequently connected to subsequent cognitive strategy use. Further, the difference between the two HPK groups was smaller than that observed among LPK learners.

### 3.3. Effects of Integrated Metacognitive Support and Prior Knowledge on Learning Performance

To address the third research question, factorial ANOVAs were conducted to examine the effects of integrated metacognitive support and prior knowledge on learners’ learning outcomes. Means and standard deviations for learning outcomes in all groups are presented in Table 4.

Table 5 presents the HC4-robust Type III ANOVA results for the learning outcome variables. Overall, integrated metacognitive support showed significant main effects on all learning outcomes, indicating that learners in the support condition achieved higher retention, comprehension, overall post-test scores, and proportional learning gains than those in the control condition. Prior knowledge also showed significant main effects on retention, comprehension, and overall post-test scores, suggesting that HPK learners generally performed better on these outcomes. However, prior knowledge was not significantly associated with proportional learning gains.

The interaction between intervention and prior knowledge was significant for comprehension and overall post-test scores, marginally significant for retention, and not significant for proportional learning gains. Follow-up simple effects analyses showed that, for comprehension, the effect of intervention was not significant among HPK learners, estimate = 0.59, SE = 0.51, *t*(99) = 1.16, *p* = 0.249, whereas among LPK learners, the intervention significantly improved comprehension scores, estimate = 2.40, SE = 0.67, *t*(99) = 3.61, *p* < 0.001. A similar pattern was found for overall post-test scores. Among HPK learners, the effect of intervention was not significant, estimate = 0.84, SE = 0.70, *t*(99) = 1.20, *p* = 0.232, whereas among LPK learners, the intervention significantly improved overall post-test scores, estimate = 3.53, SE = 0.90, *t*(99) = 3.91, *p* < 0.001. These findings suggest that the intervention improved all learning outcomes, with stronger effects on comprehension and overall post-test scores for LPK learners.

## 4. Discussion

This study explored the effects of integrated metacognitive support and prior knowledge on learners’ SRL strategies, SRL process patterns, and learning performance during hypermedia learning. In the following sections, we discuss these findings and consider their implications for the design of hypermedia learning environments.

### 4.1. Impact on Self-Regulated Learning Strategy Use

With respect to the first research question, the results revealed that integrated metacognitive support had a significant positive effect on SRL strategies. This finding is consistent with previous studies (Azevedo et al., 2016; Bannert & Reimann, 2012). An important feature of the present intervention is that it combined pre-learning metacognitive support with embedded support for planning, monitoring, reflection, and feedback during learning. This may help explain why the intervention improved learners’ use of both cognitive and metacognitive strategies. In this sense, the findings support the view that integrated metacognitive support may be more effective when knowledge and skills are addressed together (Bannert & Reimann, 2012; Donker et al., 2014). In contrast, HPK and LPK learners did not differ significantly in the frequency of coded SRL strategies, although elaboration showed a marginal trend. This finding is somewhat inconsistent with previous research (Moos & Azevedo, 2008, 2009). One possible explanation is that differences between HPK and LPK learners were reflected in how these strategies unfolded during learning. This interpretation is further supported by the ONA findings reported in Section 3.2, where prior knowledge differences emerged more clearly in SRL process patterns. The interaction between intervention and prior knowledge was also not significant for SRL strategy frequencies. This result differs from Trevors et al. (2014), who focused on the more specific behavior of note-taking. In the present study, the coded strategies were broader: note-taking was included in organization, and monitoring included several activities such as feeling of knowing and judgment of learning. Thus, differences in specific behaviors may be masked when they are grouped into broader strategy categories.

### 4.2. Impact on Self-Regulated Learning Process Patterns

The second research question focused on differences in SRL process patterns. Among LPK learners, the intervention and control groups showed clearly different process patterns. In the control condition, learners showed a reading-centered pattern, in which reading remained the central activity and was frequently followed by cognitive strategies such as rehearsal, elaboration, and organization, while monitoring functioned mainly as a temporary checkpoint before learners returned to further reading rather than making strategic adjustments. This pattern is broadly consistent with Taub et al. (2014), who found that HPK learners engaged in metacognitive strategies before cognitive strategies, whereas LPK learners engaged in cognitive strategies before metacognitive strategies.

By contrast, among LPK learners who received the intervention, monitoring became more prominent and was more closely connected with subsequent cognitive strategy use. The subtraction network further suggests that the intervention group showed stronger links among rehearsal, organization, and elaboration, indicating more flexible use of cognitive strategies to process the material. In addition, after linking new material to prior knowledge through elaboration, learners more often evaluated their understanding, monitored their learning progress, and then used rehearsal as follow-up responses. This pattern showed a more adaptive form of regulation, in which monitoring was more often followed by subsequent strategy adjustment. This interpretation is in line with earlier work showing that metacognitive support can change the sequential structure of SRL processes and promote stronger integration between cognitive and metacognitive learning activities (Sonnenberg & Bannert, 2015), as well as with theoretical accounts of SRL in which monitoring is expected to inform later strategy selection and adjustment (Winne & Hadwin, 1998).

For HPK learners, the control group showed process patterns that were closer to those observed among LPK learners who received the intervention. This suggests that HPK learners already displayed a more adaptive regulatory pattern in which monitoring was connected with subsequent cognitive strategy use, which is consistent with prior research (Taub et al., 2014). At the same time, the two HPK groups showed broadly similar process patterns. The subtraction network suggests that the intervention mainly strengthened the prominence of monitoring and its links to subsequent strategy use, rather than changing the overall process pattern. 

### 4.3. Impact on Learning Performance

The third research question examined the effects of integrated metacognitive support and prior knowledge on learning performance. The results showed that integrated support had significant positive effects on all four learning outcomes. This finding is consistent with previous studies showing that metacognitive support can improve achievement in hypermedia environments (Bannert et al., 2015; L. Guo, 2022; Zheng, 2016). The effect on proportional learning gains is particularly meaningful because this measure takes initial differences in prior knowledge into account and therefore provides a more direct indicator of learning progress during the task. The fact that the intervention remained significant for this outcome suggests that its benefits were not simply due to learners’ existing knowledge. Prior knowledge also had significant main effects on retention, comprehension, and overall post-test scores, but not proportional learning gains. This pattern is consistent with the meta-analysis by Simonsmeier et al. (2022), which showed that prior knowledge is strongly associated with post-test performance, but only weakly associated with normalized learning gains on average.

For comprehension and overall post-test scores, the intervention was more beneficial for LPK learners than for HPK learners. From an SRL perspective, LPK learners may have difficulty processing new information while also regulating their learning, because both activities require working memory resources (Winne, 2001). Instructional support, such as detailed feedback provided as a form of external monitoring, may reduce learners’ need to monitor their own progress and thereby free working memory resources for learning (Moos & Azevedo, 2008). In the present study, integrated metacognitive support may have served a similar regulatory function by guiding LPK learners’ monitoring and regulation during learning, which in turn contributed to better learning performance. This difference in learning performance was also consistent with the process findings. Among LPK learners, the intervention supported a more adaptive regulatory process during learning. 

### 4.4. Limitations and Further Research

Several limitations should be considered in future research. First, the sample was relatively homogeneous, and most participants were female. This may limit the generalizability of the findings. Future research should examine whether similar results can be found in more diverse samples and educational backgrounds. Second, the intervention was tested only in a laboratory setting. Although this allowed for greater control over the learning process, future studies should examine whether similar effects can be observed in more naturalistic learning contexts. Third, prior knowledge was divided into high and low groups using a median split. Although this approach facilitated group comparison, it may have reduced variability in the data and oversimplified individual differences in prior knowledge. Future research could treat prior knowledge as a continuous variable. Fourth, the learning interface was relatively dense and included multiple information and support components, which may have increased learners’ visual and cognitive demands. Although participants were introduced to the learning environment through a video before the formal task, the interface complexity may still have influenced their regulatory processes. Future studies could examine whether more streamlined interface designs reduce this burden while preserving the benefits of metacognitive support. Finally, SRL was assessed using coded think-aloud protocols and screen-recorded behavioral traces. While these methods yield rich process data, the present study focused on the frequency and occurrence of strategies rather than their quality. Future research should therefore complement process analyses with indicators that more directly reflect strategy quality.

## 5. Conclusions

This study examined the effects of integrated metacognitive support and prior knowledge on learners’ SRL and learning outcomes during hypermedia learning. The results showed that the support increased the use of both cognitive and metacognitive strategies and improved learning outcomes. The process analyses provided additional information by showing how SRL activities unfolded during learning. For LPK learners, the support helped them shift from a reading-centered pattern toward a more adaptive regulatory pattern, in which monitoring was more closely followed by cognitive strategy use. This may have helped these learners regulate their learning more effectively. For HPK learners, the support did not greatly change the overall process pattern, but it strengthened an existing pattern. These findings suggest that the effects of metacognitive support depend partly on learners’ prior knowledge, both in learning outcomes and in SRL process patterns. For this reason, learners’ prior knowledge should be considered when designing metacognitive support for hypermedia learning environments.

## Figures and Tables

**Table 1:** Self-regulated learning codes.

Code	Description	Examples
Planning	Activities involving goal formulation, strategy selection, activation of prior knowledge, and recycling task goals in working memory.	“First, I’ll look around to see the structure of the environment and then I’ll go to specific sections of the circulatory system.”
Monitoring	Includes judgment of learning, feeling of knowing, self-questioning, content and goal evaluation, monitoring progress and strategy use, assessing information adequacy and task difficulty, and locating information within the environment.	“I don’t really understand this; it’s difficult for me.”
Reading	Reading learning materials presented in the hypermedia environment.	“The heart is a hollow muscular organ responsible for pumping blood throughout the body.”
Rehearsal	Repeating or revisiting previously accessed sections of the hypermedia environment.	“I’m reading this part again.”
Elaboration	Elaborating on information that has been read, seen, or heard by connecting it with prior knowledge.	“After inspecting a picture of the major valves of the heart, the learner states ‘So that’s how the systemic and pulmonary systems work together’.”
Organization	Organizing information from hypermedia content, including external (visual) organization, such as distinguishing important from unimportant information through highlighting or underlining, as well as extracting, summarizing, and integrating key information or ideas.	“Highlighting or underlining key terms on the screen, such as left ventricle, left atrium, right ventricle, and right atrium, while identifying the basic structural relation that the atria are upper chambers and the ventricles are lower chambers.”
Test	Actions involving answering or completing questions embedded in the hypermedia learning environment.	“Submitting an answer after reading a passage or watching a video, such as clicking an option or typing a response in an embedded quiz.”

**Table 2:** Means (standard deviations) for SRL strategies in all groups (*N* = 99).

Variable	Control		Support	
	LPK (*n* = 29) *M* (*SD*)	HPK (*n* = 20) *M* (*SD*)	LPK (*n* = 25) *M* (*SD*)	HPK (*n* = 25) *M* (*SD*)
Planning	7.86 (2.71)	7.80 (2.80)	10.24 (3.85)	10.76 (2.09)
Monitoring	17.90 (9.31)	20.25 (7.22)	32.60 (8.05)	33.00 (7.57)
Rehearsal	7.34 (6.16)	8.05 (4.70)	12.24 (9.35)	14.36 (8.60)
Elaboration	12.79 (8.62)	19.20 (9.27)	21.52 (9.66)	22.04 (7.97)
Organization	17.24 (8.68)	18.50 (9.74)	23.72 (8.93)	24.92 (9.70)

**Table 3:** HC4-robust Type III ANOVA results for SRL strategy variables.

Variable	Source	*df*	*F*	*p*	*ηp* ^2^
Planning	Intervention	1	20.103 ***	<0.001	0.175
	Prior knowledge	1	0.148	0.701	0.002
	Interaction	1	0.239	0.626	0.003
Monitoring	Intervention	1	71.858 ***	<0.001	0.419
	Prior knowledge	1	0.723	0.397	0.007
	Interaction	1	0.364	0.548	0.004
Rehearsal	Intervention	1	14.162 ***	<0.001	0.125
	Prior knowledge	1	0.900	0.345	0.009
	Interaction	1	0.226	0.636	0.002
Elaboration	Intervention	1	10.165 **	0.002	0.098
	Prior knowledge	1	3.646	0.059	0.038
	Interaction	1	2.633	0.108	0.027
Organization	Intervention	1	11.610 **	0.001	0.111
	Prior knowledge	1	0.422	0.518	0.005
	Interaction	1	0.000	0.988	0.000

Note. ** *p* < 0.01. *** *p* < 0.001.

**Table 4:** Means (standard deviations) for learning outcomes in all groups (*N* = 103).

Variable	Control		Support	
	LPK (*n* = 32) *M* (*SD*)	HPK (*n* = 20) *M* (*SD*)	LPK (*n* = 26) *M* (*SD*)	HPK (*n* = 25) *M* (*SD*)
Retention	7.53 (1.76)	8.75 (1.02)	8.65 (1.16)	9.00 (1.26)
Comprehension	8.75 (3.18)	11.05 (1.73)	11.15 (1.83)	11.64 (1.63)
Overall post-test scores	16.28 (4.27)	19.80 (2.19)	19.81 (2.53)	20.64 (2.46)
Proportional learning gains	0.55 (0.30)	0.67 (0.21)	0.80 (0.15)	0.80 (0.21)

**Table 5:** HC4-robust Type III ANOVA results for learning outcome variables.

Variable	Source	*df*	*F*	*p*	*ηp* ^2^
Retention	Intervention	1	7.125 **	0.009	0.059
	Prior knowledge	1	9.262 **	0.003	0.076
	Interaction	1	2.880	0.093	0.025
Comprehension	Intervention	1	12.781 ***	<0.001	0.098
	Prior knowledge	1	11.069 **	0.001	0.086
	Interaction	1	4.691 *	0.033	0.038
Overall post-test scores	Intervention	1	14.684 ***	<0.001	0.110
	Prior knowledge	1	14.581 ***	<0.001	0.110
	Interaction	1	5.558 *	0.020	0.045
Proportional learning gains	Intervention	1	20.028 ***	<0.001	0.154
	Prior knowledge	1	1.775	0.186	0.016
	Interaction	1	1.712	0.194	0.015

Note. * *p* < 0.05. ** *p* < 0.01. *** *p* < 0.001.

## Data Availability

The data presented in this study are available on reasonable request from the corresponding author.
